# Impact of Bottom-Up Cocreation of Nursing Technological Innovations: Explorative Interview Study Among Hospital Nurses and Managers

**DOI:** 10.2196/60543

**Published:** 2025-03-31

**Authors:** Saskia van Steenis, Onno Helder, Helianthe S M Kort, Thijs van Houwelingen

**Affiliations:** 1 Nursing Sciences, program in Clinical Health Sciences University Medical Center Utrecht, Utrecht University Utrecht The Netherlands; 2 Department of Create4Care, Erasmus MC University Medical Center Rotterdam Rotterdam The Netherlands; 3 Research Centre Innovations in Care Rotterdam University of Applied Sciences Rotterdam The Netherlands; 4 Research Group Technology for Healthcare Innovations, Research Centre for Healthy and Sustainable Living University of Applied Sciences Utrecht Utrecht The Netherlands; 5 Building Healthy Environments for Future Users Group, Department of the Built Environment Eindhoven University of Technology Eindhoven The Netherlands

**Keywords:** stakeholder participation, cocreation, nursing, innovation, bottom-up approach, diffusion of innovation, qualitative research, nurses

## Abstract

**Background:**

In health care, the use of nursing technological innovations, particularly technological products, is rapidly increasing; however, these innovations do not always align with nursing practice. An explanation for this issue could be that nursing technological innovations are developed and implemented with a top-down approach, which could subsequently limit the positive impact on practice. Cocreation with stakeholders such as nurses can help address this issue. Nowadays, health care centers increasingly encourage stakeholder participation, which is known as a bottom-up cocreation approach. However, little is known about the experience of nurses and their managers with this approach and the innovations it results in within the field of nursing care.

**Objective:**

This study aims to explore nurses’ and their managers’ experiences with a bottom-up cocreation approach in order to assess the impact of this way of working and the resulting nursing technological innovations in an academic hospital. This insight can also inform decisions on whether the bottom-up cocreation approach should be more widely disseminated.

**Methods:**

A qualitative study using semistructured interviews was conducted with 15 participants, including cocreator nurses, end-user nurses, and their managers. First, the data were thematically analyzed. In addition, a strengths, weaknesses, opportunities, and threats analysis was conducted.

**Results:**

The various experiences of the participants were described in 3 main themes: enhanced attractiveness of the nursing profession, feeling involved due to a cocreation environment, and experienced benefits and challenges in using cocreated products. In addition, numerous strengths and opportunities perceived by the participants were identified as associated with the bottom-up cocreation approach and resulting useful products within nursing care; for example, cocreation contributed to job satisfaction and substantially contributed to the ease of use of the innovations that were developed.

**Conclusions:**

The findings underscore that cocreation with nurses enhances the appeal of the nursing profession and aligns nursing technological innovations with practical nursing challenges. Embracing a culture of cocreation has the potential to foster a culture of continuous improvement and innovation in nursing care.

## Introduction

### Background

Nowadays, technological products and other innovations are being used more frequently in the health care sector [1-3] to minimize physical strain, such as lifting patients or transporting beds, and encourage ergonomic approaches during nursing tasks. Nurses need to use these innovations during patient care to manage various health care challenges, such as the increasing demand and complexity of health care [4,5]. An example of a technological product, which will be referred to as a “nursing technological innovation” in this paper, is the TrulyEasy clamp, designed for convenient 1-handed adjustment of the arterial line to the correct height [3]. Another example of a technological product is LineConnector, a product designed to efficiently organize infusion lines and prevent tangling [3].

Unfortunately, technological solutions aimed at solving nursing issues do not always align with daily nursing practice [2,6]. A potential explanation for this lies in the top-down approach used during the development and implementation of many innovations. Policy makers may prioritize regulatory and payment interests, while technicians focus on the functionality of innovations, without fully understanding the user perspectives and the nursing context [6-8]. Consequently, some solutions may inadequately align with the needs of the nursing profession, leading to limited impact on nursing practice [2,6]. Improving the adoption of innovations in nursing practice can be addressed in the development and implementation phase of these innovations through stakeholder participation [1,5,9-12].

A growing number of health care centers are facilitating an environment that allows stakeholders to participate in the development and implementation of nursing technological innovations [1,6,9,10,12]. This involvement, also called cocreation [13], with a bottom-up approach [8], involves an interaction in which various stakeholders work together to produce an outcome that is valued by all stakeholders involved [13]. The bottom-up approach allows stakeholders to share their knowledge or opinions about issues presented in daily activities and suggest potential solutions [8,14]. This approach can support the development of nursing technological innovations that may have an impact on nursing care [1,5,9-12]. FlushEgg is an example of a cocreated nursing technological innovation. Nurses reported experiencing challenges and discomfort while flushing the narrow central lines in neonates. Therefore, a syringe attachment was designed to enhance comfort in the palm during the application of pressure [3]. Another example of a cocreated nursing technological innovation is KoosGuard, which includes a holder that can be placed in the bed through which the cables and infusion lines are guided without becoming tangled or pinched [3].

However, the bottom-up approach requires nurses to invest time, which is challenging considering many nurses face time constraints [15,16]. If these nursing technological innovations lack the required quality, this could result in decreased application in nursing practice [2,6]. Furthermore, a poorly developed nursing technological innovation could lead to resistance from nurses, resulting in a negative attitude toward these innovations [9,17]. To fully use the potential of innovations in nursing practice, the innovations need to be adequately designed, which requires the involvement of nurses [9,18]. Despite the increasing involvement of nurses in the cocreation of nursing technological innovations, little is known about how this approach and its resulting innovations are experienced by nurses. Therefore, this study seeks to explore the experience resulting from the bottom-up approach of cocreation with nurses and the resulting nursing technological innovations developed through this approach. The perspectives of nurses, both as cocreators and end users, and their managers are key to understanding the impact of this collaborative process. These results could offer a deeper insight into how nurses, both as cocreators and end users, along with their managers, perceive the bottom-up cocreation approach and its resulting nursing technological innovations. A comprehensive understanding of their experiences, both positive and negative, can significantly contribute to the overarching goal of enhancing patient care and nursing practice, making it safer and more effective.

### Aim

This study explores nurses’ and their managers’ experiences with a bottom-up cocreation approach in order to assess the impact of this way of working and the resulting nursing technological innovations in an academic hospital. This insight can also inform decisions on whether the bottom-up cocreation approach should be more widely disseminated.

## Methods

### Study Design

This study used an exploratory, descriptive, qualitative research design using individual interviews conducted at an academic hospital in the Netherlands between February and April 2023. This design facilitated the exploration of participants’ experiences, which were further analyzed through a thematic analysis and subsequently through a strengths, weaknesses, opportunities, and threats (SWOT) analysis [19]. The methods and subsequent results were reported according to the Consolidated Criteria for Reporting Qualitative Research (COREQ) checklist [20].

### Population and Setting

The study population consisted of three subgroups: (1) nurses with experience in cocreation, (2) nurses with experience as end users, and (3) health care managers from the nursing departments at an academic hospital where the innovations were implemented. In addition, all eligible nurses and managers were required to have at least 1 year of experience within the nursing department where the innovations were implemented. The 1-year experience criterion ensured that nurses and managers were sufficiently familiar with the innovations and context. The setting comprised an innovation department established within the academic hospital.

### Sampling Strategy and Recruitment

To gain more understanding and in-depth information from a broader perspective, a heterogeneous group of nurses and their health care managers were included through purposive sampling [19,21,22]. Potential nurse cocreators and managers for this study were recruited and informed via an email by OH about participating in the interviews. Interested individuals were required to actively contact SvS by email. Upon receiving an email, SvS responded with a participant information letter and an informed consent form. Once participants agreed to participate in the study, interviews were scheduled at a mutually convenient time. For the nurse end users, SvS visited the nursing departments where nursing technological innovations were integrated into nursing practice. Interested nurse end users were able to participate in an interview during their shifts at a time convenient for them. The nurse end users were informed via a participant information letter. After informed consent was given, the interviews started. The face-to-face interviews were conducted at the academic hospital.

### Sample Size

A total of 14 to 20 participants were required to obtain saturation of knowledge with a heterogeneous sample group [19]. Therefore, the aim of this study was to include at least 14 participants [19]. The study involved 15 participants, including 10 (67%) women and 5 (33%) men.

### Data Collection

Data were collected through semistructured interviews using an interview guide based on literature and expert opinions [19]. The interview guide covered various topics (Table 1), including the participants’ attitude [23] toward technology, the bottom-up cocreation approach, and the nursing technological innovations developed through this approach. It also explored the acceptance [9,10,23], usability [12,23], and adoption [10,23] of these innovations in nursing care as well as their impact on patient care and nursing practice [1,11,12,24]. Demographic characteristics (age, gender, and specialization), along with the adoption rate, provided an overview and background information of the participants in this study. The adoption rate proposed by Rogers [25] allowed the measurement of individuals’ attitudes toward innovations, providing valuable insights into how the participants generally perceive and approach innovations. The adoption rate according to Rogers [25] was measured by asking the participants which category they identified with. They could choose one of the following five categories: (1) driven by change and introduction of innovations, (2) leading in adopting innovations, (3) deliberately adopting innovations, (4) adoption is an economic necessity and a response to social peer pressure, and (5) have a traditional view and are more skeptical about innovations [25]. A pilot test was conducted to refine the interview guide and involved expert assessment and field testing with one participant. After the interviews, the first author created memos recording thoughts that arose due to the interviews [19]. The interviews were audio recorded and conducted by SvS, a nurse with no prior experience at the hospital where the research took place. SvS was trained in conducting interviews. Throughout the data collection process, discussions were held with the research team, including investigators SvS, TvH, and OH, to refine the interview questions and ensure that they effectively addressed the research question.

**Table 1:** Interview guide topics and prompt questions.

Topics	Prompt questions for nurses (end users)	Prompt questions for cocreator nurses	Prompt questions for managers
Attitude	What are your impressions regarding the fact that a coworker from your department contributed to this nursing technological innovation? What is your opinion on dedicating time to enable nurses to create and execute nursing technological innovations for nursing care?	Can you elaborate on how the co-creation process began and how it evolved? What is your opinion on dedicating time to enable nurses to create and execute nursing technological innovations for nursing care?	What are your thoughts on the fact that a nurse from the nursing department has made a substantial contribution to this nursing technological innovation? What is your opinion on dedicating time to enable nurses to create and execute nursing technological innovations for nursing care?
Acceptance	What were your initial impressions of the nursing technological innovation when you first began working with it?	What were your initial impressions of the nursing technological innovation when you first began working with it?	What responses did you observe from nurses when they were introduced to the nursing technological innovation for the first time?
Adoption	In what ways do you integrate the nursing technological innovation into your daily nursing tasks and responsibilities?	In what ways do you integrate the nursing technological innovation into your daily nursing tasks and responsibilities?	What are the viewpoints within the department regarding the utilization of the nursing technological innovation?
Usability	What is your experience with the nursing technological innovation at work?	What is your experience with the nursing technological innovation at work?	What are the experiences of the nurses regarding the use of the nursing technological innovation?
Nursing practice	What transformations do you observe in nursing practice as a result of the application of the nursing technological innovation?	What transformations do you observe in nursing practice as a result of the application of the nursing technological innovation?	What transformations do you observe in nursing practice as a result of the application of the nursing technological innovation?
Patient care	What changes do you perceive in patient outcomes as a result of the application of the nursing technological innovation?	What changes do you perceive in patient outcomes as a result of the application of the nursing technological innovation?	What changes do you perceive in patient outcomes as a result of the application of the nursing technological innovation?

### Data Analysis

The semistructured interviews were transcribed verbatim [19]. A 6-step thematic analysis approach (Table 2) was applied using ATLAS.ti software (version 23; Lumivero) [26]. Open coding and constant comparison techniques were used during the analysis [19]. An iterative process was used between data collection and data analysis. Refining the interview questions made the collection of the data more focused and specific as the process developed [19]. Audio recordings were transcribed, read, and reread by SvS in order to become familiar with the data. Data were initially coded using open coding by SvS. The 1st, 3rd, 6th, and 10th transcripts were coded separately, both by SvS and TvH. The initial codes of SvS and TvH were compared and discussed until consensus was reached. Main themes were generated through an iterative process, including constant comparison, and further elaborated and finalized by SvS, TvH, and OH. A codebook was used for insights into coding and code development [19].

**Table 2:** Thematic analysis.

Phase	Explanation
Phase 1: familiarization with the data	SvS conducted interviews with the participants and transcribed them. Transcripts were read and reread by SvS to familiarize herself with the data and obtain an overall impression.
Phase 2: generation of initial codes	Data were extracted from the transcripts and initially coded using open coding by SvS. The 1st, 3rd, 6th, and 10th transcripts were coded separately, both by SvS and TvH. The initial codes of SvS and TvH were compared and discussed until consensus was reached. The memos were processed systematically. Codes remained linked to the transcriptions (quotes) and the data extracts, using ATLAS.ti (Lumivero).
Phase 3: broader levels of themes	SvS developed broad potential main themes by comparing the initial codes. During meetings with SvS, TvH, and OH, consensus was reached, resulting in potential main themes derived from the data. During follow-up meetings with SvS, TvH, and OH, a description of the potential main themes was created and deliberated upon.
Phase 4: reviewing and refining the candidate themes	SvS compared the potential main themes with the coded data extracts and the raw data (transcriptions), discussing inconsistencies with TvH and OH. This process led to the identification of the main themes, which were visualized with a thematic map. The themes were refined by SvS and peer reviewed by TvH and OH.
Phase 5: defining and naming themes	SvS revisited the data extracts to define the content of each main theme. In meetings, SvS, TvH, and OH discussed the essence of each theme and developed the overall story line, resulting in the final naming of the main themes.
Phase 6: producing the report	SvS drafted the report and included detailed examples and quotes supporting the main themes. TvH and OH provided feedback, and adjustments were made accordingly. Final recommendations were implemented in the report.

In addition to the thematic analysis, we performed a secondary analysis using the SWOT matrix. This secondary analysis was carried out to make the data more accessible for the readers and support strategic changes within an organization. The SWOT analysis has been shown to be a valid method in previous studies for facilitating data interpretation for readers and informing strategic planning and decision-making in practice [27,28]. In several meetings, detailed examples of the results, with related quotes of the main themes, were discussed and analyzed and subsequently linked to a component of the SWOT matrix.

### Rigor and Reflexivity

Different techniques were used to enhance trustworthiness [19]. The credibility of this study was established by the investigators by creating a nonjudgmental ambiance during the semistructured interviews to obtain the participants’ perspective. The chance for bias was reduced by transcribing the content of the interviews verbatim. Member checking was used to ensure the accuracy of the transcripts, with participants reviewing the transcripts to validate their thoughts and ideas [19]. The performed member check resulted in minimal textual changes to the data.

Memos were reported during data collection and analysis to support the investigation process and the thoughts that had occurred to the investigator. The conformability of the interpretation and credibility of the data were both enhanced by investigator triangulation during the data analysis and peer feedback during researcher meetings, including the investigators SvS, TvH, OH, and HSMK. Due to peer feedback, potential meanings and a wider range of perspectives were revealed. Reflexivity, ensured by the investigator’s critical view of the interview process and comments from other investigators, increased depth and improved accuracy.

A thick description was pursued to ensure the transferability of this study through the diversity of the population base, the number of participants, and the length of the interviews for imitability. To enhance the reflexivity and transparency of the investigators, a logbook was kept, detailing all changes and decisions made throughout the process of this study [19].

### Ethical Considerations

Throughout this study, the principles of the Declaration of Helsinki and the code of conduct for health research were followed [29,30]. The study was examined and approved by the medical ethics review committee of the Erasmus Medical Center for a nonmedical scientific research application (MEC-2023-0021). Participants were informed and invited to the study by OH. They received the investigator’s email and had 1 week to express interest. Nonresponders received a reminder. The investigator sent a participant information letter and informed consent upon contact. Participants could ask further questions via email. Interviews were scheduled after participants confirmed their approval. Before the interview, they provided written informed consent. The participants did not receive any compensation for their participation in the study. During the study, data collection was conducted with confidentiality, and data were processed anonymously according to the European Union General Data Protection Regulation [29]. Only the investigators SvS, TvH, OH, and HSMK had access to the source data.

## Results

### Characteristics of the Participants

The duration of the interviews ranged from 26 to 62 minutes, with an average duration of 44 (SD 10.8) minutes. Participants’ ages ranged from 20 to 67 years. Various departments, including the intensive care unit, children’s ward, and the recovery department, were represented in the study. Table 3 presents detailed demographic information, including gender, age range, department, adoption rate, number of nurses participating as cocreators and end users, and the number of health care managers.

**Table 3:** Demographic data of the participants (N=15).

Characteristics			Participants, n (%)
**Gender**			
	Women	10 (67)	
	Men	5 (33)	
**Age range (y)**			
	20-29	1 (7)	
	30-39	5 (33)	
	40-49	3 (20)	
	50-59	3 (20)	
	60-67	3 (20)	
**Department**			
	ICU^a^ (adults)	7 (47)	
	ICU (thoracic surgery and cardiac monitoring)	2 (13)	
	Center for home ventilator and respiratory disorders in children	2 (13)	
	ICU (children)	1 (7)	
	Recovery	1 (7)	
	Day treatment (children)	2 (13)	
**Adoption rate^b^**			
	Innovator^c^	10 (67)	
	Early adopters^d^	3 (20)	
	Early majority^e^	2 (13)	
	Late majority^f^	0 (0)	
	Laggards^g^	0 (0)	
**Profession**			
	Nurse (cocreator)	6 (40)	
	Nurse (end user)	5 (33)	
	Health care manager	4 (27)	

^a^ICU: intensive care unit.

^b^Adoption rate was measured by asking the participants which category they identified with.

^c^Driven by change and introduction of innovations.

^d^Leading in adopting innovations.

^e^Deliberately adopting innovations.

^f^Adoption is an economic necessity and a response to social peer pressure.

^g^Laggards have a traditional view and are more skeptical about innovations [26].

### Bottom-Up Cocreation With Nurses

#### Overview

**Figure 1:**
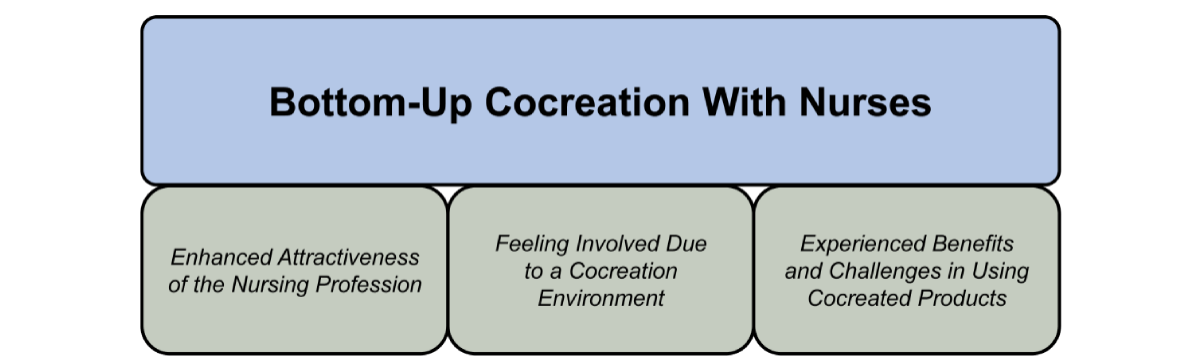
Thematic map with the 3 main themes.

The thematic analysis uncovered the following three main themes concerning the participants’ experiences with the bottom-up cocreation approach and resulting nursing technological innovations: (1) enhanced attractiveness of the nursing profession, (2) feeling involved due to a cocreation environment, and (3) experienced benefits and challenges in using cocreated products. Figure 1 provides a visual representation of the main themes.

#### Enhanced Attractiveness of the Nursing Profession

All participants experienced that the bottom-up cocreation approach with nurses contributed to the overall attractiveness of the nursing profession. The nursing profession’s attractiveness was enhanced as participants were enthusiastic about participating in cocreation, finding it enjoyable and engaging. Moreover, some participants felt that they were taken more seriously as nurses as they were the ones who actually experienced the problems in practice and were given an opportunity to use their expertise to address these challenges and drive improvements in nursing practice:

It makes work more enjoyable. You feel taken seriously as a nurse. You can share your ideas. You can use your expertise to improve something. So I think that’s a lot of fun and very inspiring, and it also leads to something useful in the end.

P11, cocreator

Some participants emphasized the importance of having affinity with cocreation. Most participants felt a sense of pride in having a cocreation department within the organization and being a part of a cocreation process. A few participants expressed modesty about contributing to the cocreation product:

Well, I think we were genuinely proud to be able to offer the product to the patient. It was a joint effort and of course it was also very important for us that our name was set out on the product. So another team could not say, “Look at this!” It really makes you feel good if you, if you can make something that you really like to use, then that is actually really nice.

P8, end user

In addition, nurses were more attracted to the profession because they assumed multiple roles during cocreation. They acted as developers among designers, students, and the nursing department, actively contributing ideas and promoting products within the departments. Some participants expressed that the opportunity to become involved in cocreation made their work more interesting, and, due to this, they did not look for employment elsewhere. Cocreation with nurses was also inspiring for some participants. It motivated other nurses to address practical problems encountered during their work and explore possibilities for solving them:

But you’re then involved in care in just a slightly different way than in standard care. And that also gives new energy and new ideas. And maybe other colleagues could also get new ideas and think, well, I have seen it a few times now, so what if I can come up with some ideas like this. Then they might think, oh well, perhaps this is something that we can also work on. This could also inspire others.

P13, cocreator

#### Feeling Involved Due to a Cocreation Environment

All participants reported enhanced collaboration through bottom-up cocreation involving various stakeholders. Collaboration included clear communication and teamwork among nurses, designers, and technically oriented students. Most participants mentioned that collaboration within the organization increased their willingness to adopt certain products that were aligned with nursing practice. One participant preferred internal product development but acknowledged that external innovations could still benefit end users:

If you get everyone involved, then you will have much more support and that is also the case with new things. I have experienced a few times in my life that we were getting an email on Sunday night. “From Monday we will do it like this.” Well, then nurses will be digging in their heels. Whereas if you say, we’re working on this, and who wants to contribute their views? Then you create support; much more people will then be willing to take it from there.

P9, cocreator

I like it when the product comes from us, I like that, but it does not necessarily have to come from us.

P8, end user

Some participants highlighted that collaboration among nursing departments, other hospitals, and organizations contributed to knowledge exchange, product distribution, and external production. However, one participant mentioned poor communication among nursing departments concerning new developments within the organization:

But we, we do note things, and we share them. It’s important that you know this. We’re not all working in separate groups.... On the contrary, a lot of information is shared among the four of us. Because we have to intervene and know what the other thinks.

P10, manager

No, that is also very poorly communicated. And the same applies to these products.

P9, cocreator

Some participants mentioned encountering practical problems but found it challenging to address these problems without the support of an innovation department with a cocreation group. The presence of such a department allowed the active participation of nurses and patients in creating specific products. Furthermore, a subgroup of participants engaged in cocreation with both patients and their parents, resulting in products tailored to their specific desires and needs. Patients were enthusiastic and felt heard, leading to the active use of end-user products:

Yes, parents have been involved in the co-creation process. That is, in the entire process of designing, they were not just allowed to give input, but were also able make direct choices, about what benefits them the most.

P12, manager

An aspect experienced by most participants was the need for support during the cocreation process. Several participants emphasized the importance of a supportive manager in facilitating and allocating dedicated time for the cocreation process. They noted that cocreation was unlikely to occur without managerial backing. Participants reported that supportive managers who recognized the value of cocreation and its potential to save time in nursing care were more proactive in addressing and resolving practical problems:

Managers can stimulate it. I think that people working in healthcare can be very creative, but often think, what shall I say, in boxes, because it’s not possible, because we’re too busy. But actually that is such a shame, because we just have a lot to offer and we have the knowledge and experience to be able to create something beautiful. And I think if a manager who (A) stimulates and (B) gives the space that is required, this will ensure that people are more motivated to contribute. Because they’re aware that improvement is possible.

P11, cocreator

#### Experienced Benefits and Challenges in Using Cocreated Products

##### Benefits

Participants found the products resulting from the bottom-up approach to be highly practical and user-friendly when used during their nursing tasks. All participants expressed immediate positivity toward the product due to its straightforward design. They could use the product without needing special training. Nurses also immediately experienced positive outcomes during their tasks. For instance, one participant mentioned that a newly created clamp for placing pressure transducers at the correct height on infusion poles improved nursing efficiency. They also reported that this clamp alleviated wrist complaints:

It is faster, yes, you press it and put it on the pole, loosen it and put it back on the transport cart.

P6 end user

Some participants found that the products improved their ability to organize and manage nursing tasks. For example, a product named KoosGuard contributed to an improved organization of patient monitoring wires, providing enhanced structure during nursing care. Another product, the cannula emergency bag, ensured that nurses, patients, and parents had all the materials neatly arranged in case of an emergency, enhancing preparedness and efficiency:

Overview yes, you immediately have a clear overview. Because it’s easy to open it, everything is in place and it’s compact. And because it was developed together with the parents and the nurses, of course, they really like that. It fits their needs.

P12, manager

Several participants emphasized the importance of the products’ quality and safety. Participants highlighted that the professional appearance of the product and its suitability for nursing practice served as indicators of quality. Participants reported that these well-suited products reduced the need for makeshift solutions, resulting in fewer inconvenient situations during their work:

Yes, the well-known example is that we often use band-aids and things, but that is to help you out for the time being. But practice has already shown that there are much more convenient solutions.

P11, cocreator

Certain participants emphasized the role of the products in enhancing patient safety. Participants noted that a specific product effectively prevented infusion line entanglement and tension, thus reducing the risk of accidental removal. In addition, participants mentioned a product designed to decrease the risk of postsurgery wound contamination, promoting improved healing outcomes:

You really want those lines to be properly arranged. To make sure that not one of them is hidden from your view, because if one of those lines is pulled, the infusion may accidentally get disconnected or the patient can accidentally end up lying on top of that line because you can’t see it.

P14, manager

##### Challenges

In addition to the benefits of the products, participants also experienced some challenges in their use. Some participants mentioned that some product designs had flaws, which hindered their effectiveness. For example, a participant heard from some patients that they still thought that the cannula emergency bag was too large to be used conveniently, and, due to this, it was no longer used. In addition, it was indicated that some products looked disposable, so nurses threw them away:

The only negative feedback came from a patient. She just thought the bag was too big.

P8, end user

A few participants pointed out that some products were prone to getting lost or becoming untraceable. It was emphasized that these products should be readily available to nurses at designated and standardized locations. Otherwise, the product will be used less or not used at all. Furthermore, some participants shared their struggle with making changes, leading to a heightened risk of reverting to old routines:

Then it takes a lot of time anyway. And you think: well, I’ll just do it in the old-fashioned way, using a gauze.

P3, end user

#### SWOT Analysis Findings

Detailed examples of the main themes were divided into the 4 components of the SWOT matrix. Multimedia Appendix 1 presents an overview of all the main themes, along with detailed examples, illustrative quotations, and the corresponding SWOT categorization. The “strengths” and “weaknesses” encompass the experienced advantages and disadvantages associated with the bottom-up cocreation approach and the products developed through this approach. The “opportunities” and “threats” are examples that could be associated with potential future opportunities and challenges of the bottom-up cocreation approach and developed products. Most of the examples were connected to the components “strengths” and “opportunities.”

## Discussion

### Principal Findings

In this study, we sought to explore hospital nurses’ experience with a bottom-up cocreation approach and the resulting nursing technological innovations developed through this approach. Notably, through a SWOT matrix, we mainly revealed positive experiences of participants cocreating nursing technological innovations, with several strengths and opportunities. Participants’ experiences can be described in 3 themes: enhanced attractiveness of the nursing profession, feeling involved due to a cocreation environment, and experienced benefits and challenges in using cocreated products. The findings indicate that participants found the nursing technological innovations easily applicable in nursing practice due to their user-friendly design, the ability to enhance nursing efficiency, patient safety, and time-saving potential during nursing work. A collaborative culture may lead to higher work satisfaction among nurses, inspire cocreation, and promote nursing pride.

### Comparison With Prior Work

This study shows how cocreating nursing technological innovations positively impacts the attractiveness of the nursing profession. The attractiveness of the nursing profession was enhanced by nurses finding joy in their roles, feeling a sense of pride, and being taken seriously in their work. This aligns with findings from a cross-sectional study, which emphasizes the significance of decision-making involvement in health care services, such as equipment, technologies, and processes for employment preferences of nursing students [31]. In addition, it aligns with a systematic review indicating that an increase in career opportunities and challenges positively impacts nurse retention. It reduces nurses’ desire to leave a particular workplace [32].

Furthermore, this study emphasizes several collaboration advances due to a cocreation environment within the organization, such as clear communication and teamwork among nurses, designers, and technically oriented students. Moreover, a cocreation environment within the organization contributed to knowledge exchange, product distribution, and external production. An in-depth case study [14] reinforced the importance of an innovative environment within the organization. It could increase the diffusion of nurse-led innovations within and outside the organization. Particularly, managers seem to have an important role in collaborating and communicating with each other about innovations and supporting working methods in nursing departments. Existing literature [33,34] reinforces the notion that supportive leadership is crucial for fostering a culture of innovation and enhancing the sustainability and impact of health care innovation settings.

In addition, this study shows that innovations cocreated with nurses led to multiple benefits for nursing practice. Cocreated nursing technological innovations were described as user-friendly, highly practical, and easy to integrate into nursing tasks, consistent with findings from a quasi-experimental study [35]. In this quasi-experimental study, trained innovative nurses were able to identify issues in nursing practice and apply creative thinking strategies to create innovative products that matched their nursing work routines. Furthermore, the cocreated nursing technological innovations met nursing quality requirements and reduced the need for improvised solutions, thereby minimizing inconvenient situations in nursing practice. Patient safety was also enhanced by the cocreated products, which prevented issues such as infusion line entanglement and reduced the risk of wound contamination. The incubator traffic light is an example from the literature of a cocreated nursing technological innovation that meets quality standards and enhances patient safety. The incubator traffic light features a visual feedback system for neonatal incubators designed to enhance hand hygiene compliance [1,2]. Incubators with the incubator traffic light visual feedback system demonstrated significantly higher compliance with the correct drying times compared to those without this feature. This could potentially enhance patient safety by reducing infection rates in neonatal intensive care units [1]. Although the cocreated nursing technological innovations offered several advantages, the study also encountered certain challenges in their use, for example, flaws in the design of the innovations, which hindered their effectiveness or ability to make changes in the nursing routines. This study shows that changes in nursing processes may increase the risk of reverting to old routines, which aligns with findings from a cross-sectional survey that indicated how changes in nursing work processes could influence established work habits [36].

While this study did not include patients or parents directly, the participants acknowledged the significance of their perspective during the innovation process. It was mentioned that some of the cocreated innovations were developed in collaboration with patients and parents, resulting in innovations aligning with their expectations and needs. This is consistent with existing literature, where patients, family caregivers, and clinicians cocreated a mobile health app for heart failure self-management tailored to the specific health care context [37]. Furthermore, it matches with a qualitative co-design article including family, physicians, researchers, patients, and industry partners. The study revealed important differences between the participants’ preference for functional requirements of a mobile health app [38].

### Limitations

Limitations of the study include its single-site focus on a specific academic hospital and limited transferability to other health care settings [19]. Furthermore, there may be a sampling bias, as interested individuals were required to actively contact the investigator via email to participate in this study. These individuals might have been more likely than other professionals to embrace the innovations and encourage their adoption within their teams. In addition, all the participants scored themselves above average on the adoption rate scale (Table 3), and 10 (67%) of the 15 participants considered themselves to be “innovators” [25]. Literature shows that only 2.5% of the population belongs to the group of innovators [25]. This suggests a potential bias in the participant group. Including nurses as cocreators may have introduced selection bias, as their enthusiasm and assertiveness toward innovations may have influenced the results in a more positive manner [39]. Finally, the sample size consisted of 6 (40%) nurses as cocreators, 5 (33%) nurses as end users, and 4 (27%) managers. The limited insights from the managers’ perspective may affect the comprehensiveness of the findings in the study [19].

### Implications and Further Research

The findings of this study suggest several implications. Health care organizations could consider adopting the bottom-up cocreation approach with nurses as it has the potential to enhance nurses’ work satisfaction, a sense of pride among nurses, and adoptable nursing technological innovations in nursing care. Furthermore, cocreation can contribute to accessible collaborations between stakeholders inside and outside the organization. In addition, cocreation with nurses has the potential to facilitate the development of innovations that enhance the efficiency of nursing work. Simple, professional, and time-saving innovations created through this approach could improve nursing practices.

Further research is recommended to explore the impact of the bottom-up cocreation approach and the resulting nursing technological innovations in other health care centers, aiming to improve the transferability of this way of working. Moreover, to better address patients’ expectations and needs during the cocreation process and resulting innovations, further research is recommended to investigate their experiences throughout the cocreation process and its outcomes.

### Conclusions

This study highlights substantial positive experiences of cocreating nursing technological innovations in nursing care. The findings underscore that cocreation with nurses enhances the appeal of the nursing profession. Participants perceived cocreation as enjoyable, leading to heightened work satisfaction and a sense of pride in their nursing role. Moreover, cocreation with nurses fosters the development of nursing technological innovations that align with nursing practice challenges, thereby facilitating their adoption within nursing care. Ultimately, embracing a culture of cocreation has the potential to foster ongoing improvements and innovations in nursing care, further contributing to the professionalization of the field.

## Appendix

Multimedia Appendix 1 Main themes; detailed examples; illustrative quotes; and strengths, weaknesses, opportunities, and threats categorization.

## Abbreviations

COREQ: Consolidated Criteria for Reporting Qualitative Research
SWOT: strengths, weaknesses, opportunities, and threats

## Data Availability

The datasets generated or analyzed during this study are not publicly available due the participants involved in this research did not authorize the public dissemination of their data through written consent.
