# Supplementary material for: Impact of Bottom-Up Cocreation of Nursing Technological Innovations: Explorative Interview Study Among Hospital Nurses and Managers
Source: JMIR Hum Factors. 2025 Mar 31;12:e60543. doi: 10.2196/60543 (PMC11997522; doi:10.2196/60543)
Supplement: Multimedia Appendix 1 [file humanfactors_v12i1e60543_app1.docx]

**Multimedia Appendix.** Main themes; detailed examples; illustrative quotes; and strengths, weaknesses, opportunities, and threats (SWOT) categorization.

| Main themes and detailed examples | | Illustrative quotations | Categorization according to SWOT |
| --- | --- | --- | --- |
| **Enhanced attractiveness of the nursing profession** | | | |
|  | Pride of cocreation within the nursing profession | “But also just that you are proud that you have achieved something, yes, whatever other people use now and that they are happy with the product. And that it provides an advantage for yes, for the colleagues who use it.” [P.13, cocreator] | Strength |
|  | Cocreation contributed to job satisfaction | “Yes, I really, really enjoyed working on the co-creation process.” [P.7, cocreator] | Strength |
|  | Nurses want to be involved during the creation of new developments | “Yes, you need to inform people, communicate, gather them together, indicate when the product will come, and establish its usefulness and necessity.” [P.12, manager] | Opportunity |
|  | Problems identified in practice by nurses | “People were also very pleased with the product, partly because they also ran into the practical problem themselves. So they just thought it was like, hallelujah, so something is going to change. So yes, that was very motivating and people were happy about it.” [P.11, cocreator] | Strength |
|  | Listing practical problems encountered by nurses | “Sometimes it is just the very small things that can be so useful. And yes, I think so, yes, definitely, because we used to have an idea box, you know. Then you could…that is very important…Let everyone contribute their views, what would you like and what is feasible of course. But just think about what you would like. How something should work. And then you will automatically find out whether it is feasible or not. But I think a lot more is possible than many people think, I think.” [P.8, end user] | Opportunity |
|  | Fulfill an additional role as a nurse cocreator | “Well, we really developed it as a team. It was not just me. I was the person that mediated between the students and the nursing team.” [P.7, cocreator] | Opportunity |
|  | Cocreation inspires other nurses | “What we see is that nurses like the co-creation process. That they notice where gaps still exist, where improvements are possible. And there are a number of nurses who like this so much that we also give them time and space for it. And you notice that sometimes there are almost too many things that are brought forward. That we actually have make a selection. That is what happens in the department. So, it is stimulating. In the department, they are bringing it more and more into the spotlight, so you see it more often.” [P.12, manager] | Opportunity |
|  | Retaining nurses | “That applies to, for example, innovations that contribute to one’s physical capacity. So, for example, a cart such as that, for a dialysis machine, where you no longer have to lift so many bags because they can just be slid into such a cart. This means that people, so to speak, get fewer physical complaints and are therefore longer employable and remain more vital. So in the end, you hope to make a profit. Yes, profit meaning that people just want to continue their nursing work. That it remains attractive.” [P.14, manager] | Opportunity |
|  | Nurses’ affinity to cocreate | “Yes, the concept of co-creation is of course completely fine. So I am really one hundred percent behind that. Only do you notice, of course, that there’s a group that always says ‘yes, but everything was better in the old days.’ So because as a nursing group you always have to go forward, you always keep pulling those people along, so to speak. They’re less keen about innovations or change and say, ‘why not keep doing it in the way we are used to?’ So that is an issue but it’s good that you can contribute your ideas. But I am not sure whether every nurse likes to contribute in that way.” [P.2, end user] | Opportunity |
|  | Nurses’ modesty about contribution to cocreation | “But at first they also wanted my name. Then I said no, it is not just me, I think that is too much credit. So we also chose to make it the name of the nursing department.” [P.7, cocreator] | Weakness |
| **Feeling involved due to a cocreation environment** | | | |
|  | Cocreation teamwork ensures support for new products | “I think co-creation is a good thing. Because if it comes from the work floor, from the bedside, that means that the implementation is also better. Yes, look, you hope it will be worn by the people standing at the bedside. That the ones that are working at the base say ‘guys, can we improve this,’ it’s good if the need is expressed within the group. You can say top-down: ‘this is what we are going to do and this is an improvement,’ but it is just […] that has less chance of succeeding than when you get it from the workplace, from the basics. Like, ‘this looks like a bottleneck, it’s really bothering us.’ Then, try to find out how this can be improved and what could be a solution.” [P.10, manager] | Opportunity |
|  | Cocreation department within the organization for the fit of the products | “The product is what is most desirable and what is most optimal for parents and nurses. This encourages enthusiasm. And, of course, there are parents who already have years of experience with a child using a cannula. So they have already tried different things. They are looking for products that are the most convenient. And they can’t find them. Then suddenly there’s something is actually tailor-made. And then the circle is completed and there you have it.” [P.12, manager] | Opportunity |
|  | Distribution of the products through communication between departments | “And then I think: I only hear positive reactions and also other nursing departments that sometimes need these diapers. They now come and get them from us.” [P.11, cocreator] | Opportunity |
|  | Sharing new developments with other hospitals and organizations | “So I believe when you say ‘from practice’ yes, but then I also believe in a kind of networking, where you can create a place where people can come together to share and develop the ideas.” [P.15, manager] | Opportunity |
|  | Explorative market research | “Well, I have been looking around for a long time myself, thinking about where we might procure any such items. But they just weren’t available.” [P.9, cocreator] | Opportunity |
|  | Cocreation department needed within the organization | “So, within an organization, an innovation department is essential to facilitate such advancements. Otherwise, it would become extremely difficult to reach such goals.” [P.1, cocreator] | Opportunity |
|  | Involvement of patients (and their parents) during the creation of new developments | “But patients focus more on the practical side of things. They say things like ‘I thought that one was very clever, that handle or that infusion line connection is not convenient, it gets loose very quickly,’ you know, that sort of things.” [P.8, end user] | Opportunity |
|  | Supportive manager increases cocreation | “If the manager is enthusiastic about something. Then you see that the nurses will seek opportunities. If managers lack enthusiasm and are not allowing space...Then nothing happens.” [P.12, manager] | Opportunity |
|  | Facilitating cocreation increases cocreation | “Look, of course managers are removed at quite a distance from the workplace. Well, that may also vary considerably per manager. But I only saw my manager for a brief moment in the mornings and then she just went into meetings. So in practice, I don’t think you can expect them to say ‘well you have to do something with this.’ So not at that level, but managers could facilitate it and promote it, yes, yes.” [P.11, cocreator] | Opportunity |
|  | Saving time by investing nursing time to cocreate | “Because if you can use such a simple plastic thingy to ensure that those lines are always snugly arranged or don’t drag over over the floor, transport becomes easy. Then that time is eventually quickly recouped.” [P.15, manager] | Opportunity |
| **Experienced benefits and challenges in using cocreated products** | | | |
|  | Ease of use product | “But otherwise it is of course a very simple product, which is just very practical and just enjoyable for us. Because we like it when things are tight and in order and not tangled, ha-ha.” [P.2, end user] | Strength |
|  | Product immediately clear | “The product is not complicated technology, shall I say, or an entire device, for example. It is very simple. It is easy to operate, you can understand it immediately. Just one instruction and you know how it works.” [P.5, cocreator] | Strength |
|  | Product relevant | “The product is not something that we just like and then is not used anymore, but it is still being used daily. Yes, so it’s fully integrated into the nursing care.” [P.13, cocreator] | Strength |
|  | Product provides overview | “I see that there’s something about the product that has made it easier for parents to take the materials with them in an orderly and arranged manner. Instead of it being tossed into a bag and you just have to hope that you can find it at the right moment. So it’s really improved I think.” [P.7, cocreator] | Strength |
|  | First positive reaction to products | “Yes, enthusiastic, they just saw, when she used it, that there was an improvement like: it works quickly, easy, no longer any need to turn it around.” [P.5, cocreator] | Strength |
|  | Product is time saving | “Yes, normally those lines get all tangled up and get stuck in the rails. And now with that thing, you can put everything through it. So that helps a lot to make sure it does not get tangled up and things like that. And I think it is really wonderful that it is so simple. It is just fantastic. You just click, put your lines through it and you’re set.” [P.2, end user] | Strength |
|  | Ergonomic benefits for nurses | “You just notice especially with the product of [name co-creator], we used to be standing there moving the thing up and down. Yes, and your wrists got injured. And now, you can squeeze it and put it up and down. And that is, that just saves a lot of work and a lot of physical strain.” [P.3, end user] | Strength |
|  | Professional appearance of the product | “Yes, only this product looks more professional.” [P.4, end user] | Strength |
|  | Professionalization of nursing products | “Well, yes, before this, you could fabricate something yourself of course. You could just take a card and then tie all the lines together and tie them to the cushions.” [P.3, end user] | Opportunity |
|  | Product meets quality requirements | “Yes, easy to clean, yes, that of the assistants, our care assistants who also clean the room when the patient is gone. And with the old thing, you know, you have a thread. And if tube feeding gets in between or infusion fluid…that leaves a residue. That is a lot harder to remove. This new surface is completely smooth, so uh, easier to clean.” [P.5, cocreator] | Strength |
|  | Supports preventive patient safety | “Yes, that clamp is quite handy, because you always have the bundle of lines together. Yes, and as I also said, there is often less tension if you put it on it because then...Otherwise, for example, it will drop between the bed and the bed rail. And then it gets stuck there or and you know, so can you prevent that kind of things from happening if you use that clamp.” [P.2, end user] | Strength |
|  | Patient unaware of the product | “Most people are sedated. They do wake up, but are often not fully conscious. Or yes, you know prepared for surgery. The next day they are awake, but they do not notice this product.” [P.5, cocreator] | Strength |
|  | Flaws in design | “So what happens then, the infusion pump hangs here, and then the chamber of the infusion pump is still not hanging straight. So then it only worked if the pump was not hanging in between.” [P.9, cocreator] | Weakness |
|  | Product gets lost or is untraceable | “It is very easy to take the product off the bed and put it back on. Which is also a disadvantage I think, because of course I also lost track of it sometimes.” [P.14, manager] | Weakness |
|  | Old routines | “But the people who have been working here for 20 years or 25 years, yes, they have found a way of working, were they say, well, this works. It is very difficult to make them change their ways.” [P.10, manager] | Threats |
